# PE_PGRS3 ensures provision of the vital phospholipids cardiolipin and phosphatidylinositols by promoting the interaction between *M. tuberculosis* and host cells

**DOI:** 10.1080/21505594.2021.1897247

**Published:** 2021-03-23

**Authors:** Flavio De Maio, Alessandro Salustri, Basem Battah, Ivana Palucci, Federica Marchionni, Silvia Bellesi, Valentina Palmieri, Massimiliano Papi, Eliza Kramarska, Maurizio Sanguinetti, Michela Sali, Rita Berisio, Giovanni Delogu

**Affiliations:** a Dipartimento di Scienze di Laboratorio e Infettivologiche, Fondazione Policlinico Universitario “A. Gemelli”, Rome, Italy; b Dipartimento di Scienze biotecnologiche di base, cliniche intensivologiche e perioperatorie – Sezione di Microbiologia, Università Cattolica del Sacro Cuore, Rome, Italy; c Dipartimento di Diagnostica per Immagini, Radioterapia Oncologica ed Ematologia, Fondazione Policlinico Universitario “A. Gemelli”, IRCCS, Rome, Italy; d Dipartimento di Neuroscienze, Università Cattolica del Sacro Cuore, Roma, Italy; e Fondazione Policlinico Universitario “A. Gemelli”, Rome, Italy; f Institute of Biostructures and Bioimaging - CNR-IBB, Naples, Italy; g Mater Olbia Hospital, Olbia, Italy

**Keywords:** PE_PGRS, phosphatidylinositols, adhesion, host interaction, tuberculosis

## Abstract

PE_PGRS proteins of *Mycobacterium tuberculosis* (*Mtb*) constitute a large family of complex modular proteins whose role is still unclear. Among those, we have previously shown, using the heterologous expression in *Mycobacterium smegmatis*, that PE_PGRS3 containing a unique arginine-rich C-terminal domain, promotes adhesion to host cells. In this study, we investigate the role of PE_PGRS3 and its C-terminal domain directly in *Mtb* using functional deletion mutants. The results obtained here show that PE_PGRS3 is localized on the mycobacterial cell wall and its arginine-rich C-terminal region protrudes from the mycobacterial membrane and mediates *Mtb* entry into epithelial cells. Most importantly, this positively charged helical domain specifically binds phosphorylated phosphatidylinositols and cardiolipin, whereas it is unable to bind other phospholipids. Interestingly, administration of cardiolipin and phosphatidylinositol but no other phospholipids was able to turn-off expression of *pe_pgrs*3 activated by phosphate starvation conditions. These findings suggest that PE_PGRS3 has the key role to serve as a bridge between mycobacteria and host cells by interacting with specific host phospholipids and extracting them from host cells, for their direct integration or as a source of phosphate, during phases of TB pathogenesis when *Mtb* is short of phosphate supply.

## Introduction

*Mycobacterium tuberculosis* (*Mtb*) is one of the most successful human pathogens, that co-evolved with humans for more than 100.000 years [1–3]. The most common outcome following *Mtb* infection is latent tuberculosis (TB), a benign coexistence between the human host and the tubercle bacilli that usually last for a lifetime, with no signs or symptoms of disease [4,5]. In 2–8% of cases, *Mtb* infection leads to overt disease, which most often involves the lung tissue, with inflammation, caseous necrosis, cavitation, and extensive tissue damage, that allows for the aerogenic transmission of *Mtb* [6,7]. Hence, *Mtb* usually survives and resists in host tissues for a lifetime despite the presence of a robust innate and adaptive immune responses. We lack a clear understanding of the immunological mechanisms involved in this process and molecular determinants responsible for these unique features of *Mtb* have been only partially identified [8].

Proteins and other molecules available on the mycobacterial surface and mycomembrane are known to play a key role in host–pathogen interaction [9]. Among these, some of the ESX type 7 secretion systems (T7SS) are essential for *Mtb* virulence, with the secretion of effector proteins such as those of the EsxA/B family and PE and PPE family [10]. The expansion and diversification of PE and PPE proteins was a key event in the evolution of the virulent *Mtb*, where these genes occupy almost 10% of the genome coding capacity [11–13]. Some of these proteins, as in the case of the PE_PGRS subfamily, are found only in the *Mtb* complex and few other pathogenic mycobacteria affecting mammals, where they have been implicated in mycobacterial pathogenesis [14].

In *Mtb*, there are more than 50 functional *pe_pgrs* genes which encode proteins with a shared structure: a highly conserved PE domain of ≈ 100 amino acids in length; a linker domain with a conserved GRPLI motif that is essential for proper protein translocation on the mycobacterial outer membrane; a polymorphic glycine-rich domain of variable size with repetitive gly-gly-X motif [15,16]. Some of the PE_PGRS proteins have a unique C-terminal domain that can be up to 300 amino acids in length as for PE_PGRS30 [17]. A mounting body of experimental evidence is supporting the role of PE_PGRS proteins in TB pathogenesis [14,18]. We have recently shown that PE_PGRS3 of *Mtb* contains, downstream of the PGRS domain, a unique arginine-rich C-terminal domain, with 30 arginine units out of the distal 77 amino acids [19]. In a series of experiments involving the heterologous expression of the *pe_pgrs*3 gene in *Mycobacterium smegmatis* (*Ms*), we have shown that PE_PGRS3 is specifically expressed under low phosphate concentrations and mediates adhesion to host cells through the arginine-rich domain [19]. In this study, we provide clues of the functional role of the arginine-rich domain of PE_PGRS3 in experimental models involving the use of *Mtb*. Our findings suggest that PE_PGRS3 plays an essential role in grabbing essential components of *Mtb*, whose provision needs to be ensured for mycobacterial survival.

## Results

### The arginine-rich C-terminal domain of PE_PGRS3 enhances mycobacteria entry in host cells

To investigate the role of the arginine-rich C-terminal domain of the PE_PGRS3 (xR-3Ct), the sequence corresponding to the 240 nucleotides at the 3ʹ of the *Rv0278c* gene, coding the xR-3Ct domain of 80 amino acids, was cloned in pET-SUMO vector and expressed in *E. coli* using standard procedures [20] (Supplementary Figure 1a **–**d). Following purification, dialyzed and endotoxin-free recombinant xR-3Ct was used in a series of experiments to assess its functional role. Murine macrophages (J774) and human alveolar epithelial cells (A549) were infected with a panel of *Ms* recombinant strains with and without the addition in the culture medium of the recombinant xR-3Ct and adhesion and cell entry assessed by intracellular bacterial counting 4 h post-infection.

**Figure 1.**
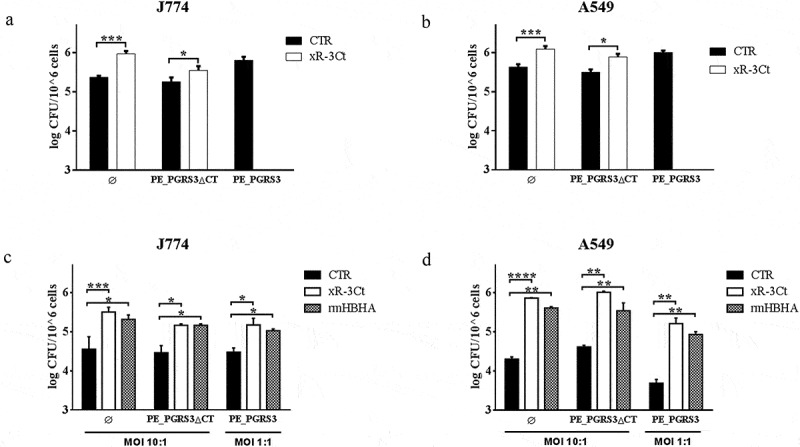
Recombinant arginine-rich C-terminal domain of the PE_PGRS3 promotes *Mycobacterium smegmatis* adhesion to macrophages and pneumocytes

Murine macrophages (J774, panel **A**) and human type 2 pneumocytes (A549, panel **B**) were infected (MOI 10:1) with recombinant *Mycobacterium smegmatis* (*Ms*) expressing cytosolic green fluorescent protein (*Ms^GFP^*, indicated as Ø), or co-expressing GFP and full-length *pe_pgrs3* gene (*Ms^GFP^PE*_*PGRS3^HA^*, indicated as PE_PGRS3) and its functional mutant lacking the C-terminal domain (*Ms^GFP^PE_PGRS3∆CT^HA^*, indicated as PE_PGRS3ΔCT). Dialyzed LPS-free PE_PGRS3 C-terminal domain (xR-3Ct) was added to the infection solution at a final concentration of 5 µg/ml when both cell types were infected with *Ms^GFP^* and *Ms^GFP^PE_PGRS3∆CT^HA^*. Four hours post infection colonies forming units (CFUs) were determined. xR-3Ct promoting adhesion was compared with that of the recombinant mycobacterial heparin-binding hemagglutinin (rmHBHA). J774 (**C**) and A549 (**D**) were infected with *Ms^GFP^PE_PGRS3^HA^, Ms^GFP^PE_PGRS3∆CT^HA^* and *Ms^GFP^*. MOI 10:1 was used for the *Ms^GFP^PE_PGRS3∆CT^HA^* and *Ms^GFP^*, whereas MOI 1:1 was used for the *Ms^GFP^PE_PGRS3^HA^*. LPS free xR-3Ct and rmHBHA were added to the infection solutions at a final concentration of 5 µg/ml. Four hours post infection CFUs were determined. CFUs are reported in log10 scale and represented as mean ± SD and comparisons were obtained by using Two-way ANOVA with Tukey’s correction.

The addition of the recombinant xR-3Ct protein to a strain of *Ms* expressing the green fluorescent protein (*Ms^GFP^*) enhanced adhesion to macrophages and pneumocytes, to achieve efficiency in cell entry similar to those observed for the recombinant *Ms* strain expressing PE_PGRS3 under the control of a strong promoter (*Ms^GFP^PE_PGRS3^HA^*) [19] (Figure 1a **and b**). Interestingly, the addition of the recombinant xR-3Ct complemented the *Ms* expressing the functional mutant of the PE_PGRS3 lacking the C-terminal domain (*Ms^GFP^PE_PGRS3ΔCT^HA^*) (Figure 1a **and b**).

To better characterize these findings, J774 and A549 cells were infected using a lower MOI (1:1) of the *Ms^GFP^PE_PGRS3^HA^* and compared with the infection carried out with the other functional deletion mutant strain and the parental strain administered at a higher MOI (MOI = 10:1) (Figure 1c **and d**). As expected, since the *Ms^GFP^PE_PGRS3^HA^* was administered at a ten times lower dose, the intracellular CFUs measured following infection were similar in J774 and in A549 with the CFUs of the other strains in non-conditioned media. However, when the recombinant xR-3Ct protein was added to the media, we observed enhanced mycobacterial entry in macrophages and pneumocytes for the three strains, regardless of the MOI used (Figure 1c **and**d).

Moreover, we observed the same phenotype when we added another polybasic protein as the mycobacterial HBHA, which is known to mediate adhesion to epithelial cells but not to macrophages [21,22]. Again, no differences between *Ms^GFP^* and recombinant *Ms* expressing PE_PGRS3 or its chimeras were observed, although, in contrast to previous findings [22] addition of rmHBHA enhanced mycobacterial entry also in macrophages. These discrepancies may be attributable to the different experimental settings used and we cannot exclude that the addition of rmHBHA may activate macrophages to enhance bacterial uptake [23,24].

***The PE_PGRS3 arginine-rich C-terminal domain specifically binds phosphatidylinositols and cardiolipin.***

**Figure 2.**
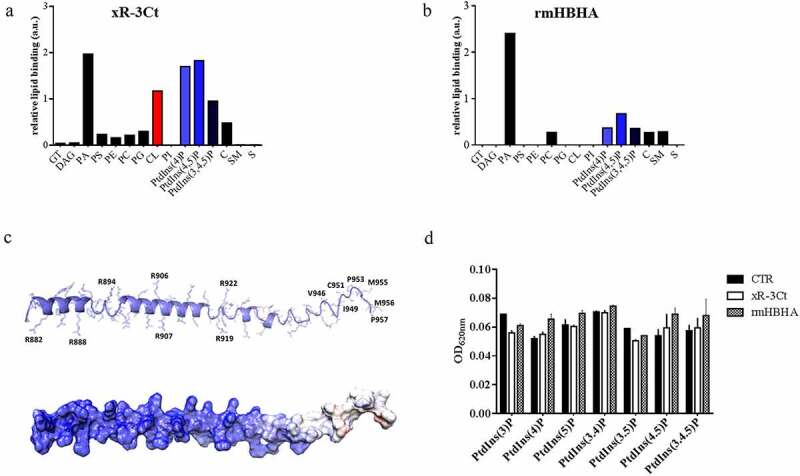
Arginine-rich C-terminal domain specifically binds cardiolipin and phosphorylated phosphatidylinositols

Polybasic proteins may interact with host components through binding to negatively charged molecules such as phospholipids commonly found in host membranes. Indeed, a recent report indicates that the lysine-rich domain of the HBHA specifically binds to 4,5 di-phosphorylated phosphatidylinositol [25]. To investigate the ability of the PE_PGRS3 to bind phospholipids, the purified recombinant xR-3Ct was used to probe a nitrocellulose membrane where a series of phospholipids were adsorbed as described in the material and methods section (Supplementary Figure 2a **and b**). Following incubation, the anti-His antibody was used to detect the xR-3Ct protein captured by adsorbed lipids. The recombinant methylated HBHA (rmHBHA) was used as a positive control [25,26]. As shown in Figure 2a, recombinant xR-3Ct was able to bind phosphorylated phosphatidylinositol-4 phosphate (PtdIns (4)P), phosphatidylinositol-4,5 phosphate (PtdIns 4,5(P)), and phosphatidylinositol-3,4,5 phosphate (PtdIns (3,4,5)P), with affinity two times higher than the rmHBHA (Figure 2a **and b**). Despite the clear binding to phosphorylated phosphatidylinositol, neither xR-3Ct nor rmHBHA are able to bind phosphatidylinositol (PI). Surprisingly, recombinant xR-3Ct showed a specific interaction with cardiolipin (CL). Hence, these two polybasic mycobacterial proteins show differential ability to bind common phospholipids found in host membranes.

Purified PE_PGRS3 positive charged C-terminal (xR-3Ct) was probed on a nitrocellulose membrane where different negatively charged phosphorylated lipids were adsorbed (**A**). Mycobacterial heparin-binding hemagglutinin (rmHBHA), which shows positive charged lysine amino acidic residues, was used as control (**B**). After the incubation, monoclonal anti-His antibody was used to detect the “baits” lipids that linked recombinant proteins. Secondary HRP-antibody was used to detect the binding and each membrane signal was acquired maintaining unvaried exposition. xR-3Ct showed a significant binding with PtdIns(4)P, PtdIns 4,5(P) and PtdIns (3,4,5)P compared to rmHBHA. Conversely, both proteins bound the PA. Single spots were analyzed by using *ImageJ* software. TG: Triglyceride, DAG: Diacylglycerol, PA: Phosphatidic acid, PS: Phosphatidylserine, PE: Phosphatidylethanolamine, PC: Phosphatidylcholine, PG: Phosphatidylglycerol, CL: Cardiolipin, PI: Phosphatidylinositol, PtdIns(4)P: Phosphatidylinositol 4 phosphate, PtdIns 4,5(P): Phosphatidylinositol 4,5 phosphate, PtdIns (3,4,5)P: Phosphatidylinositol 3,4,5 phosphate, C: Cholesterol, SM: Sphingomyelin, S: Sulfatide. Secondary structure prediction of xR-3Ct and electrostatic potential surface were obtained using JPRED and Chimera software as described in Materials and methods section (**C**). xR-3Ct phosphatase activity was probed on a series of phosphorylated PtdIns substrates. Free inorganic phosphate was measured by using a malachite green assay (**D**). xR-3Ct and rmHBHA showed similar and not significant dephosphorylation activity against PtdIns. Data are represented as mean ± SD and comparisons were obtained by using Two-way ANOVA with Tukey’s correction.

The analysis of chemical structures of all tested phospholipids allows rationalization of these results. Indeed, phosphatidic acid, phosphorylated phospholipids, and cardiolipin present a stronger overall negative charge (from −2 to −7), whereas all other phospholipids are either neutral or they present a single negative charge (Supplementary Figure 3). A strong electrostatic feature in the recognition of phospholipids by xR-3Ct well agrees with its predicted structural features. Structurally, xR-3Ct is predicted to adopt an extended α-helical conformation, followed at its C-terminus by a coiled-coil region (Figure 2c). Analysis of the electrostatic potential of this molecule shows a strongly positively charged surface for the helical region, whereas the coiled-coil region presents a neutral charge, with a hydrophobic character at its C-terminus (GISCSOQMMP) (Figure 2c). Therefore, it is not surprising that xR-3Ct recognizes amphipathic molecules, with the highest avidity for those with a higher negative charge.

**Figure 3.**
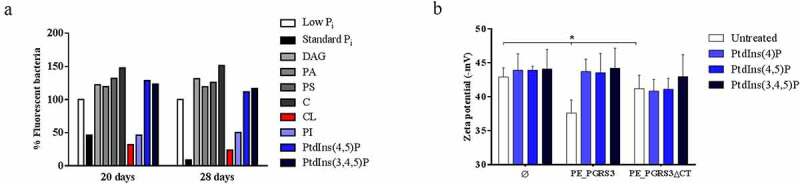
Administration of cardiolipin, but not phosphorylated phosphatidylinositols, to phosphate-starved culture turns off *pe_pgrs*3 expression

Recombinant *Mycobacterium smegmatis* (*Ms*) expressing PE_PGRS3 under control of its promoter fused at the C-terminal with the GFP (*Ms*PE_PGRS3^GFP^), *Ms* expressing GFP (*Ms*^GFP^) and *Ms* mc^2^ 155 were grown in standard and low phosphate Sauton medium (~50 μM P_i_). The fluorescence emission following expression of the GFP was measured flow cytometry as described in the Materials and Methods section. When *Ms*PE_PGRS3^GFP^ showed a significant fluorescence in low P_i_ medium, the liquid culture was split into different tubes. Inorganic phosphate (P_i_), diacylglycerol (DAG), phosphatidic acid (PA), phosphatidylserine (PS), cardiolipin (CL), phosphatidylinositol (PtdIns), PtdIns(4,5)P2, PtdIns(3,4,5)P3, and cholesterol were added at the final concentration of 100 µM in different prepared tubes. The fluorescence was measured at different time points following phosphate or lipid restoring (**A**). Data were normalized to fluorescence measured for *Ms*PE_PGRS3^GFP^ cultured in low P_i_ environment (100% of fluorescent bacteria). *Ms* over-expressing full-length PE_PGRS3 (*Ms^GFP^PE*_*PGRS3^HA^*, indicated as PE_PGRS3), its functional mutant lacking the C-terminal domain (*Ms^GFP^PE_PGRS3∆CT^HA^*, indicated as PE_PGRS3ΔCT) and *Ms* parental strain (*Ms^GFP^*, indicated as Ø) were selectively incubated with PtdIns(4)P, PtdIns 4,5(P) and PtdIns (3,4,5)P. Two hours later Zeta potential was measured (**B**). *Ms^GFP^PE_PGRS3^HA^* net surface charge modification was observed after incubation with all selected PtdIns, shifting from less negative values up to values measured for the *Ms^GFP^PE_PGRS3∆CT^HA^* and *Ms^GFP^*. Data are represented as mean ± SD and comparisons were obtained by using Two-way ANOVA with Tukey’s correction.

To corroborate the hypothesis that xR-3Ct acts solely by sequestering phospholipids and has no phosphatase activity, the recombinant xR-3Ct was incubated with different phosphorylated PtdIns and the phosphatase activity was determined by measuring free phosphates (P_i_) in solution with a malachite green assay. As shown in Figure 2d, no release in P_i_ was observed following incubation, thus showing that binding of phospholipids occurs without phosphatase activity.

***Administration of free phosphate, cardiolipin and phosphatidylinositol to phosphate-starved culture turns off pe_pgrs3 transcriptional activity.***

Since *pe_pgrs*3 is specifically expressed in phosphate starvation environment [19], we investigated the consequences on the *pe_pgrs*3 transcriptional activity in a culture of phosphate starved *Ms* expressing PE_PGRS3-GFP under the control of the *Mtb pe_pgrs*3 promoter (*Ms*PE_PGRS3^GFP^), following the administration of phosphorylated and non-phosphorylated host lipids. Phosphate starvation triggers the expression of PE_PGRS3-GFP and administration of inorganic phosphate turns off GFP expression, as previously described (Figure 2c) [19]. As expected, administration of non-phosphorylated lipids as cholesterol (C) and diacylglycerol (DAG) did not turn off *pe_pgrs*3 expression, but rather further induced fluorescence in the *Ms*PE_PGRS3^GFP^ phosphate starved culture (Figure 3a). Interestingly, administration of cardiolipin (CL) and phosphatidylinositol (PI) to *Ms*PE_PGRS3^GFP^ phosphate starved culture resulted in the turn off of fluorescence, suggesting that the mycobacterial cells do not any longer need the function of PE_PGRS3 in these conditions.

The highest level of fluorescence turn-off was observed upon the addition of free phosphate (Figure 3a). Conversely, PtdIns (4,5)P and PtdIns (3,4,5)P administration did not induce loss of fluorescence, suggesting that under these conditions (that is at least in *Ms*) these phosphorylated lipids cannot provide phosphate to the mycobacterial cells. This result prompted us to investigate whether binding by the C-terminal domain of the PE_PGRS3 to PtdIns (4)P, PtdIns 4,5(P), and PtdIns (3,4,5) is also observed with the whole bacterial cells. To this aim, we incubated *Ms* cells, cultured until mid-log phase, and resuspended in water, with the panel of selected PtdIns and then measured the Z-potential of mycobacteria. As shown in Figure 3b, no significant differences were observed between the surface charges of the *Ms^GFP^PE_PGRS3ΔCT^HA^* and *Ms^GFP^* strains, whereas *Ms^GFP^PE_PGRS3^HA^* is characterized by a significantly lower Z potential (Figure 3b). Incubation of the *Ms^GFP^PE_PGRS3^HA^* strain with the phosphorylated PtdIns enhanced the Z potential until the full retrieval of the net surface charge of the parental strain. Specifically, Zeta potential measurements showed that *Ms^GFP^PE_PGRS3^HA^* net surface charge shifted from – 37.6 ± 1.9 mV to values of –43.7 ± 1.8 mV, –43.5 ± 2.8 mV, and – 44.2 ± 2.9 mV after the incubation with PtdIns(4)P, PtdIns 4,5(P) and PtdIns (3,4,5)P, respectively. Conversely, Z potential values of *Ms^GFP^PE_PGRS3ΔCT^HA^* did not significantly change upon incubations with the phosphorylated PtdIns. These findings confirm our previous observation (Figure 2a) that the xR-3Ct domain specifically interacts with phosphorylated PtdIns. Altogether, these data suggest that cardiolipin and PI may serve as a source of phosphate for mycobacteria, through the action of specific mycobacterial phosphatases. On the other hand, the inability of the phosphorylated PtdIns 4,5(P) and PtdIns (3,4,5) to turn off *pe_pgrs*3 transcriptional activity, albeit being able to bind the xR-3Ct domain, may be ascribed to the possible lack in *Ms* of specific phosphatases able to hydrolyze and free the phosphate ions from these more complex molecules.

***Over-expression of the full-length PE_PGRS3 improves adhesion of the Mtb to pneumocytes, but not to phagocytic cells.***

**Figure 4.**
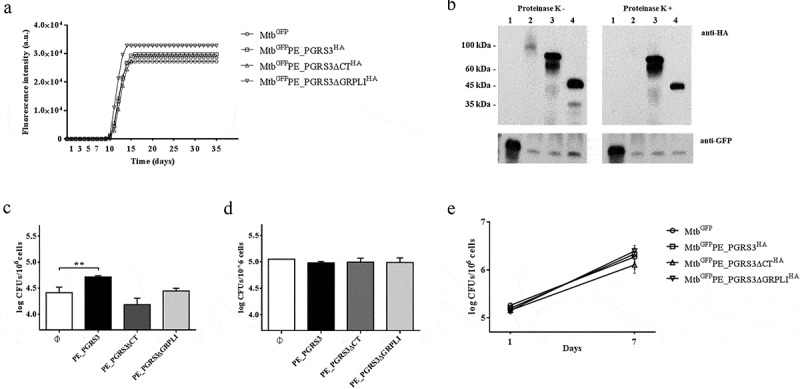
Over-expression of the PE_PGRS3 enhances *Mycobacterium tuberculosis* cell entry in pneumocytes, but not in murine macrophages or human peripheral blood mononuclear cells (PBMCs)

To investigate the functional role of the PE_PGRS3 in TB, we over-expressed various functional variants in *Mtb*, including (i) the full-length PE_PGRS3, (ii) its functional mutant lacking CT domain (residues 878–957, *Mtb^GFP^PE_PGRS3ΔCT^HA^*) and (iii) its further truncated mutant, also lacking a part of the PGRS domain (residues 528–957, *Mtb^GFP^PE_PGRS3ΔGRPLI^HA^*). Over-expression of the PE_PGRS3 in *Mtb*, or of its functional mutants, did not affect bacterial morphology (data not shown) nor growth rate in liquid media (Figure 4a). Proteinase K experiments show degradation of PE_PGRS3, thus suggesting that in *Mtb* the PE_PGRS3 full-length protein localizes on the mycobacterial surface (Figure 4b). Conversely, the two functional mutants were protected from proteinase K treatment, as proteinase K induced no degradation (Figure 4b). This finding indicates that these protein regions are not completely available on the mycobacterial surface and that the observed degradation of full-length PE_PGRS was limited to the C-terminal domain, which carries the HA epitope for detection. Namely, these results suggest that the full-length PE_PGRS3 is likely embedded in the mycomembrane and exposes solely the C-terminal arginine-rich domain.

*Mycobacterium tuberculosis* (*Mtb*) H37Rv was electroporated to over-express PE_PGRS3, and its functional mutant under the control of *hbha* gene promoter, together with the green fluorescent protein (GFP) under the control of the *Ag85b* promoter. *Mtb^GFP^PE*_*PGRS3^HA^*, expressing the full-length gene, *Mtb^GFP^PE_PGRS3ΔCT^HA^* and *Mtb^GFP^PE*_*PGRS3ΔGRPLI^HA^*, expressing the PE_PGRS3 without the last 80 amino acids (xR-3Ct) and last 430 amino acids (PGRS domain starting from the second GRPLI motif), respectively, were generated. An *Mtb* expressing GFP under the control of the *hsp60* promoter was used as control (*Mtb^GFP^*). (**A**) Bacterial growth rate of *Mtb*^GFP^, *Mtb*^GFP^PE_PGRS3^HA^, *Mtb*^GFP^PE_PGRS3∆CT^HA^, and *Mtb*^GFP^PE_PGRS3∆GRPLI^HA^ performed in Mycobacteria Growth Indicator Tube (MGIT). Measured fluorescence intensity (a.u.) and days of culture were reported to obtain the growth profile. (**B**). *Mtb^GFP^PE_PGRS3^HA^, Mtb^GFP^PE_PGRS3ΔCT^HA^,* and *Mtb^GFP^PE_PGRS3ΔGRPLI^HA^* and the parental strain *Mtb*^GFP^ were grown in Sauton medium until mid-log phase, when cells were collected. Proteinase K protection assay was performed before obtaining immunoblots of the whole cell lysates. Membranes were probed with anti‐HA monoclonal and anti-GFP polyclonal antibodies. Lanes 1, 2, 3, and 4 represent whole cell lysates of *Mtb*^GFP^, *Mtb^GFP^PE_PGRS3^HA^, Mtb^GFP^PE_PGRS3ΔCT^HA^,* and *Mtb^GFP^PE*_*PGRS3ΔGRPLI^HA^*, respectively. xR-3Ct was observed surfaced exposed. Bacterial entry was evaluated by infecting human alveolar epithelial cells (A549) (**C**) and murine macrophages (J774) (**D**) with previously mentioned recombinant strains at MOI 10:1 and MOI 1:1, respectively. Colonies forming units (CFUs) were evaluated 1 h post-infection. *Mtb*^GFP^, *Mtb^GFP^PE_PGRS3^HA^, Mtb^GFP^PE_PGRS3ΔCT^HA^,* and *Mtb^GFP^PE_PGRS3ΔGRPLI^HA^* were here indicated as Ø, PE_PGRS3, PE_PGRS3ΔCT, and PE_PGRS3ΔGRPLI, respectively. A significant result was observed for *Mtb^GFP^PE_PGRS3^HA^* recombinant strain infecting A549 (*p < 0.01*), but not in J774. Healthy donor peripheral blood mononuclear cells were isolated and infected at MOI 1:10. CFUs were evaluated at 1 and 7 days post infection (**E**). *Mtb^GFP^PE_PGRS3^HA^* did not exert increased entry or persistence compared to *Mtb*^GFP^, *Mtb^GFP^PE_PGRS3ΔCT^HA^,* or *Mtb^GFP^PE_PGRS3ΔGRPLI^HA^*. Data are represented as mean ± SD and comparisons were obtained by using one-way ANOVA is used to assay significance and CFUs are reported in log10 scale.

To investigate the effect of this exposed xR-3Ct domain on *Mtb* adhesion and entry in host cells, we infected pneumocytes (A549) and macrophages with the recombinant *Mtb* strains and evaluated the intracellular CFUs 1-h post infection. The *Mtb^GFP^PE_PGRS3^HA^* strain showed a superior entry ability in A549 (Figure 4c) but not in J774 macrophages (Figure 4d) compared to the parental strain *Mtb^GFP^*. Conversely, the *Mtb^GFP^PE_PGRS3ΔCT^HA^* and the *Mtb^GFP^PE_PGRS3ΔGRPLI^HA^* did not show any significant difference in their ability to gain entry in pneumocytes and macrophages. These results show that PE_PGRS3 enhances *Mtb* entry in epithelial cells but not in macrophages and that the enhanced cell entry is specifically due to the arginine-rich C-terminal domain. It is likely that *Mtb* entry in macrophages, which is mediated by multiple receptors, is already highly efficient and cannot be improved by PE_PGRS3. Indeed, infection of human peripheral blood mononuclear cells (PBMCs) with the *Mtb* strains did not result in any difference in total CFUs at day 1 and 7 post-infection among the strains, suggesting that PE_PGRS3 is not involved in the mycobacterial replication in the phagocytic cells (Figure 4e).

### PE_PGRS3 is post-translationally cleaved in Mtb

Prompted by the observation that the PE_PGRS3 C-terminal domain is exposed to proteolysis, we performed a subcellular fractionation of the *Mtb* strains under study to assess the role of the arginine-rich domain in the association of the PE_PGRS3 with the mycobacterial cell wall. Indeed, localization of PE_PGRS proteins in *M. smegmatis* is only partially informative as a model of *Mtb* [27], as *Ms* lacks the ESX-5 secretion system that is involved in the translocation/secretion of PE_PGRS proteins [28, 29].

**Figure 5.**
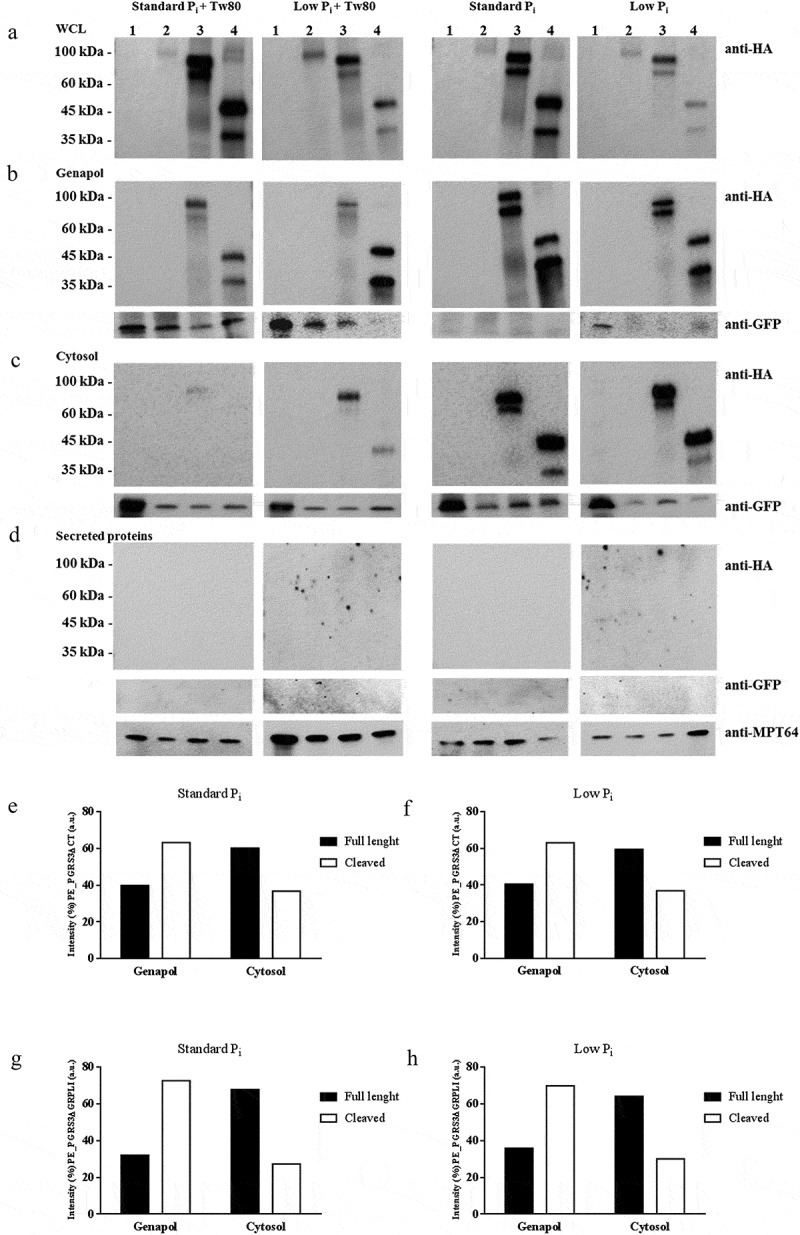
PE_PGRS3 is cleaved in *Mycobacterium tuberculosis.*

In the fractionation experiment, whole-cell lysate, the genapol extract, the cytosolic fraction following genapol extraction, and the culture supernatant (to measure secreted proteins) were obtained from the recombinant *Mtb* strains under study and cultured in Sauton media with or without Tween80 and in standard P_i_ or low P_i_ concentrations. As tested by western blot analysis, full-length PE_PGRS3^HA^ (≈ 81 kDa) was detected only in the whole cell lysate fraction of the *Mtb^GFP^PE_PGRS3^HA^* (Figure 5a), but not in any of the other fractions, regardless of the presence in the culture media of the mild detergent Tween80 or of low or standard Pi concentrations (Figure 5b**–**d). Differently, clear bands corresponding to their expected molecular weights were observed for *Mtb^GFP^PE_PGRS3ΔCT^HA^* and *Mtb^GFP^PE*_*PGRS3ΔGRPLI^HA^*, which were grown in the same conditions as the full-length PE_PGRS3. Consistent with its sensitivity to proteinase K, these findings suggest that the C-terminal domain of PE_PGRS3 is readily degraded.

*Mycobacterium tuberculosis* expressing green fluorescent protein (GFP) (*Mtb*^GFP^) or co-expressing GFP and native PE_PGRS3 (*Mtb^GFP^PE_PGRS3^HA^*) or its chimeras, lacking the xR-3Ct and part of the PGRS domain (*Mtb^GFP^PE_PGRS3ΔCT^HA^* and *Mtb^GFP^PE_PGRS3ΔGRPLI^HA^*) were cultured in different Sauton medium supplemented or not by inorganic phosphate (P_i_) or Twen80 (Tw80). Cultures were harvested at the mid-log phase (OD_600nm_: 0.8 ± 0.2) and processed to obtain a protein sub-cellular analysis. The following fractions were obtained: **A**) whole cell lysate (WCL), **B**) cell wall-associated proteins extracted after treatment with Genapol X-80 detergent, **C**) cytosolic – membrane proteins obtained after lysis of the pellet pre-treated with Genapol X-80 and **D**) secreted proteins gained by TFA – precipitation of the pre-filtered culture medium. Proteins were separated by SDS – page by using a 5–15% gradient polyacrylamide gel and then immunoblots were performed. Each membrane was probed with a monoclonal anti-HA antibody, a polyclonal anti-GFP antibody, and with an anti-MPT64 serum for the secreted protein fraction. PE_PGRS3^HA^ signal (≈81 kDa) was detected only in the WCL fraction. Clear signals were highlighted for the functional chimeras of the PE_PGRS3 (PE_PGRS3∆CT^HA^ ≈ 71 kDa) or the PE_PGRS3∆GRPLI^HA^ ≈ 45 kDa) in WCL, in the Genapol fraction, and in the cytosolic fraction. A characteristic cleavage was observed at the N-terminal of both PE_PGRS3 chimeras with resulted cleaved proteins that showed ≈ 10 kDa less than the expected molecular weight. P_i_ did not appear conditioning protein stability, whereas Tw80 significantly reduced the signal associated with the PE_PGRS3 chimeras in the Genapol fractions. Signals detected in cytosolic and genapol fractions for PE_PGRS3∆CT^HA^ and PE_PGRS3∆GRPLI^HA^, and their respective cleaved chimeras were analyzed by using ImageJ software. Densitometric analysis was carried out on *Mtb^GFP^PE_PGRS3ΔCT^HA^* (**E – F**) and *Mtb^GFP^PE_PGRS3ΔGRPLI^HA^* (**G – H**) grown in standard Sauton medium or P_i_ depleted medium.

However, the results obtained with the other two functional deletion mutants provide relevant information. Subcellular fractionation of *Mtb^GFP^ PE_PGRS3ΔCT^HA^* clearly indicates that culture in Tween80 dramatically reduces the PE_PGRS3ΔCT^HA^ extracted by Genapol or remaining in the cytosol following Genapol extraction, yet no signal was detected in the secreted fraction. Similar results were obtained with the *Mtb^GFP^ PE_PGRS3ΔGRPLI^HA^* strain, suggesting that the second GRPLI domain within the PGRS region of PE_PGRS3 does not significantly contribute to protein localization. These results indicate that growth in Tween80 containing medium significantly perturbs the localization of PE_PGRS protein on the mycobacterial surface and impacts cell permeability.

Interestingly, both PE_PGRS3ΔCT^HA^ and PE_PGRS3ΔGRPLI^HA^ were detected as two bands, with the lower band corresponding to the protein cleaved at N-terminus approximately 10 kDa downstream the amino acid in position 1. The relative amount of the lower band was higher in the genapol extract compared with cytosol obtained following genapol extraction, suggesting that cleaved PE_PGRS3 is more abundant on the mycobacterial surface (Figure 5e**–h**). Cleavage of the two proteins was consistent across the experimental conditions tested in **Figure 5** and accounted for a loss of ≈ 120–150 amino acids, with the N-terminus truncated protein stable and more abundant in the outer membrane.

### PE_PGRS33 is differently cleaved in Mtb and in *M. bovis* BCG

A recent report indicates that in *M. marinum* PE_PGRS proteins are cleaved at their N-terminus by the protease PecA, which is also present in other pathogenic mycobacteria species including *Mtb* [30]. Since we could observe the cleavage for the truncated PE_PGRS3ΔCT but not for the full-length PE_PGRS3, we decided to investigate the same process for the well-characterized PE_PGRS33 by performing the cellular fractionation of the *Mtb* and *M. bovis* BCG expressing PE_PGRS33 ^HA^. The recombinant *pe_pgrs*33 gene was expressed in *Mtb* and BCG under the control of its own promoter as previously described [31,32], which warrants constitutive expression of the protein.

**Figure 6.**
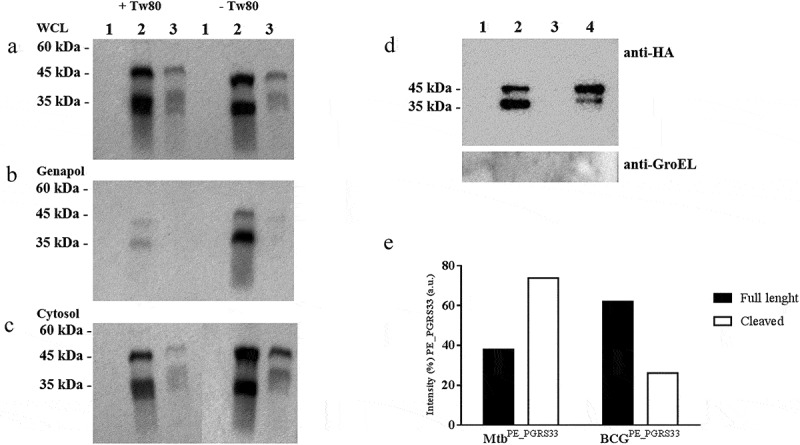
PE_PGRS33 is mainly cleaved in *Mycobacterium tuberculosis* compared to *Mycobacterium bovis* BCG

As shown in Figure 6a**–**c, PE_PGRS33 was detected at the expected molecular weight (≈45 kDa) and at a lower molecular weight (≈35 kDa), suggesting that in *Mtb* and BCG PE_PGRS33 is cleaved similarly to what observed for the PE_PGRS3-truncated proteins. Again, a much stronger signal was detected in the genapol extract of *Mtb*PE_PGRS33 ^HA^ cultured in the absence of Tween80 compared with that grown in the presence of Tween80, confirming that the presence of this mild detergent significantly alters mycobacterial permeability. The cleaved PE_PGRS33 was more abundant in the genapol extract than in the cytosolic fraction obtained following genapol extraction, suggesting that the cleaved form is most abundant in the mycobacterial cell wall. Moreover, PE_PGRS33 was poorly expressed in BCG compared with *Mtb*, despite both strains were transformed with the same plasmid, suggesting that protein stability or localization in BCG may be impaired.

*Mycobacterium tuberculosis* (*Mtb*) H37Rv and *Mycobacterium bovis* (*Mbov*) BCG, both expressing the PE_PGRS33 under the control of its putative promoter and tagged at C-terminal with the HA epitope, and the parental strain *Mtb* were grown in Sauton medium, supplemented or not with 0.05% Tween 80, until mid-log phase (OD_600nm_: 0.8 ± 0.2). Cultures were harvested at the mid-log phase (OD_600nm_: 0.8 ± 0.2) and processed to obtain a protein sub-cellular analysis. The following fractions were obtained: **A**) whole cell lysate (WCL), **B**) cell wall-associated proteins extracted after treatment of mycobacteria with Genapol X-80 detergent, **C**) cytosolic – membrane proteins obtained after lysis of the pellet pre-treated with Genapol X-80. Proteins were separated by SDS – page by using a 5–15% gradient polyacrylamide gel and then immunoblots were performed. Each membrane was probed with the anti-HA monoclonal antibody. A clear signal was detected for the full-length PE_PGRS33 (PE_PGRS33 ^HA^ ≈ 45 kDa) and its cleaved chimera (≈ 35 kDa) for both recombinant mycobacteria in WCL and in cytosolic fraction. Conversely, Genapol fractions highlighted clear signals only for the *Mtb* strain. Lane 1: *Mtb* H37Rv; Lane 2: *MtbPE*_*PGRS33 ^HA^*; Lane 3: *MbovPE_PGRS33 ^HA^*. A characteristic cleavage was observed at the N-terminal of both PE_PGRS3 chimeras with resulted cleaved proteins that showed ≈ 10 kDa less than the expected molecular weight. P_i_ did not appear conditioning protein stability, whereas Tw80 significantly reduced the signal associated with the PE_PGRS3 chimeras in the Genapol fractions. Genapol fraction of the *MtbPE_PGRS33 ^HA^* and *MbovPE_PGRS33 ^HA^* and respective parental strains were incubated with anti-HA magnetic beads. Eluates were precipitated before protein separation by SDS page electrophoresis and immunoblot (**D**). The membrane was probed with the anti-HA monoclonal antibody and anti-GroEL polyclonal antibody. Lane 1: *Mtb* H37Rv; Lane 2: *MtbPE*_*PGRS33 ^HA^*; Lane 3: *Mbov* BCG; Lane 4: *MbovPE_PGRS33 ^HA^*. PE_PGRS33 ^HA^ and cleaved chimeras-associated signals were quantified as previously mentioned (**E**). PE_PGRS33 ^HA^ cleavage was observed slightly more evident in *Mtb* than in *Mbov*BCG strain.

To further corroborate this hypothesis, the genapol fractions of *Mtb* and BCG strains expressing PE_PGRS33 ^HA^ were mixed with anti-HA antibody carried on magnetic beads to capture the protein available on the mycobacterial surface. Immunoblot of the eluted fractions clearly shows that in *Mtb* most of PE_PGRS33 is cleaved, while in BCG PE_PGRS33 is not efficiently cleaved (Figure 6d **and**e). These results support the findings that in BCG, PE_PGRS translocation on the mycobacterial cell wall is impaired [33].

## Discussion

PE_PGRS3 is a protein belonging to the PE_PGRS family endowed with a peculiar and unique arginine-rich C-terminal domain [19]. The *pe_pgrs3* gene was shown to be specifically expressed under low inorganic phosphate (P_i_) concentrations in *Mtb* and when heterologously expressed under the control of its own promoter in *Ms*. In this study, we aimed at rationalizing the role of PE_PGRS3 in connection with *Mtb* needs when in short supply of phosphate.

Using protease protection assays, we show that PE_PGRS3 is available on the *Mtb* surface, as its arginine-rich C-terminal domain is readily degraded. Consistently, subcellular fractionation studies show that PE_PGRS3 is found in the genapol extract, as expected for a protein that localizes in the mycomembrane [27,34,35b]. Importantly, the arginine-rich C-terminal domain of PE_PGRS3 protruding from the mycomembrane can promote adhesion to host cells, even when loosely associated or released by the mycobacterial cell. These results indicate that the adhesion properties of PE_PGRS3 [19] may not require a mycomembrane – bound protein as expected for an adhesin, but rather suggest that the xR-3Ct domain serves as a “bridge” between mycobacteria and host cells to promote adhesion through electrostatic interactions mediated by its arginine residues.

The role of PE_PGRS3 in mediating *Mtb* interactions with the host cells does not explain the need of *Mtb* to overexpress this protein only when phosphate is lacking. To attempt an explanation for this finding, we screened the binding of PE_PGRS3 with several phospholipids with different complexities. As a result, we found that xR-3Ct of PE_PGRS3 binds phosphorylated phosphatidylinositol (PtdIns), cardiolipin (CL), and phosphatidic acid (PA) but no other phospholipids, with a higher affinity than observed for HBHA [25]. We observed the binding of these PtdIns also to mycobacterial cells expressing PE_PGRS3, as demonstrated by the significant alterations of the Z potential of the mycobacterial surface in the presence of PtdIns. Therefore, xR-3Ct domains protruding from the mycobacterial surface mediate binding to the negatively charged lipids.

Structural features of xR-3Ct, displaying a positive electrostatic potential and a hydrophobic C-terminal arm, well account for the recognition of phosphatidylinositol, which carry both negatively charged phosphates and a lipidic arm. Similar considerations apply to cardiolipin, also able to bind xR-3Ct and to induce the turning off of the *pe_pgrs*3 transcriptional activity. These experimental evidences suggest that phosphate-starved mycobacteria start expressing PE_PGRS3 protein that then translocates to the mycobacterial surface to expose the arginine-rich domain and becomes available to bind host-derived cardiolipin and phosphatidylinositol. Lipids are known to be a major source of energy for *Mtb* in several steps of TB pathogenesis. In the caseating debris of human TB granulomas, there is an enrichment of cholesterol, cholesteryl esters, and triacylglycerides (TAGs), with the latter being the main components of the large lipid droplets commonly found in foamy macrophages, another important niche for *Mtb* [36,37]. Phagosome-containing *Mtb* can be found tightly associated with lipid droplets [38] and *Mtb* can translocate to these droplets where it utilizes lipids as a carbon source for survival [39,40]. Interestingly, elevated levels of TAGs are found during rapid disease development caused by modern *Mtb* Beijing strains compared to ancient *Mtb* Beijing strains [41], highlighting the importance of lipid metabolism in TB pathogenesis. Since these lipids lack phosphorous, the growth and persistence of *Mtb* under these nutrient conditions occurs in an environment where phosphate is a limiting factor [42]. Hence, *Mtb* must be able to deploy mechanisms aimed at stealing phosphorylated molecules from the host to maintain adequate levels of this essential component.

Cardiolipin is a phosphorylated lipid that constitutes 10–20% of the mitochondrion inner membrane [43] and damaged mitochondria release cardiolipin in the outer membrane and then in the cytoplasm. *Mtb*-infected and damaged foamy macrophages and necrotic cells that abound in the caseous granuloma may accumulate higher levels of cardiolipin released by damaged mitochondria. We hypothesize that PE_PGRS3 on the *Mtb* surface can serve to grab cardiolipin through the arginine-rich domain, protruding from the mycomembrane; cardiolipin can then be imported or degraded with the help of other proteins, to obtain phosphate. A similar scenario may be proposed for the phosphatidylinositol commonly found in the eukaryotic membranes. It is possible that *Mtb*-specific proteins are required to obtain phosphates from these phosphatidylinositols or that *Mtb* can simply accumulate these phospholipids on the surface and that host enzymes as PTEN [44] may free phosphate molecules that can then be imported by *Mtb*. The role of PE_PGRS3 as a “molecular thief” well explains its overexpression in the absence of phosphate, and by grabbing these molecules, PE_PGRS3 ensures proper provisions of phosphate ions. Further studies are needed to support this hypothesis, though the unique presence of the arginine-rich domain in *Mtb* but not in other mycobacteria highlights further interest in the role of this protein in TB pathogenesis.

Moreover, PtdIns has been demonstrated to be of vital importance for the growth and viability of mycobacteria since it acts as a lipid anchor for key components of the cell wall, like the glycolipids phosphatidylinositol mannoside (PIM), lipomannan (LM), and lipoarabinomannan (LAM). In *Mtb*, PtdIns synthesis is catalyzed by the CDP-alcohol phosphotransferase phosphatidylinositol-phosphate synthase (PIPS), an essential enzyme for mycobacterial viability [45]. Catalyzed by PIPS, PtdIns is obtained by the chemical reaction of CDP-diacylglycerol with inositol-phosphate to form phosphatidylinositol-phosphate, the precursor to PtdIns. PtdIns is unique to mycobacteria and few other bacterial species whereas it is widely distributed in eukaryotes, where PtdIns(4)P and PtdIns(4,5)P are commonly recognized to mediate anchoring of eukaryotic polybasic proteins [46] and are involved in several pathways, including key steps in the intracellular lifestyle of *Mtb* in host cells as phagosome/lysosome fusion and autophagosome formation [18,47]. The essential role of PtdIns in *Mtb*, the availability of this compound in host cells, and the avidity of xR-3Ct for PtdIns point to the hypothesis that PE_PGRS3 exerts the precise role to enhance host cell interaction and, even more important, to tear PtdIns molecules from the host cells.

Another interesting feature of PE_PGRS3, arisen from subcellular fractionation studies of PE_PGRS3ΔCT^HA^ and PE_PGRS3ΔGRPLI^HA^, is that a cleavage of the protein occurs that removes the N-terminal 120–150 residues, immediately upstream the PGRS domain. Densitometric analysis of these immunoblots clearly indicates that the cleaved protein is more abundant in the genapol extract than in the cytosolic fraction. Similar results were obtained when the same experiment was carried out in *Mtb* expressing the well-characterized PE_PGRS33. The cleavage we observed for PE_PGRS3 and PE_PGRS33 is in line with recent findings showing that in *M. marinum* LipY and PE_PGRS proteins are processed by another PE_PGRS protein, PecA [30]. Cleavage of the PE protein LipY by PecA in *M. marinum* occurs at positions 136 and 149 [48], whereas cleavage of PE_PGRS proteins results in the loss of ≈11 kDa, corresponding to the PE domain. The observed cleavage in *Mtb* is located within the position 139– 152 of PE_PGRSs, which includes, also in PE_PGRS3, the characteristic Ser-Gly–Gly–Ala motif that is the putative target of the protease PecA. Despite several efforts, we could not purify enough cleaved PE_PGRS33 or PE_PGRS3 to identify the exact position of the cleavage site.

In *M. marinum* and *Mtb*, translocation of PE_PGRS proteins, including PecA, occurs through the ESX5 type 7 secretion system [29,49,50] and cleavage of the PE domain by the protease PecA occurs on the mycobacterial surface [30]. Our findings that the cleaved forms of PE_PGRS3 and PE_PGRS33 are more abundant in the genapol extract than in the cytosolic fraction support this hypothesis, and the model we recently proposed [14]. Moreover, the observation that expression of PE_PGRS33 in BCG results in poorly cleaved protein is in line with the recent findings by Ates et al. [33,51], indicating impaired translocation of PE_PGRS proteins in BCG.

Another note that we think is relevant to briefly discuss involves the impact that the use of a nonionic detergent in the culture medium has on the mycobacterial cell. In fact, culturing *Mtb* in liquid media containing Tween80 had a relevant impact on protein localization, indicating that this mild detergent may extract PE_PGRS3, PE_PGRS33, and potentially all other PE_PGRS proteins from the mycomembrane. This is in our opinion very important since it indicates that culturing *Mtb* in the presence of Tween80 could perturb the mycomembrane, leading to a loss of surface protein. As a result, culturing mycobacteria and *Mtb* in media without Tween80 or other mild detergents is desirable when performing protein subcellular fractionation studies or other functional assays aimed at studying mycomembrane-associated proteins.

In conclusion, the results of this study suggest that PE_PGRS proteins, once translocated through the plasmatic membrane in an ESX5-dependent mechanism [29,30], remain associated with the mycomembrane in a cleaved state missing their PE domains. Once in the outer membrane PE_PGRS proteins may “float” on the mycomembrane outer leaflet and may possibly be released to exert their activity as seen for instance for PE_PGRS3 and its arginine-rich motif that can bind host membranes. Similarly, PE_PGRS33 that interacts with TLR2 may be released or freed from the outer leaflet of the mycomembrane to promote inflammation and tissue damage [31,32]. We expect a similar scenario for other PE_PGRS proteins known to play a role during the intracellular lifestyle of *Mtb* in macrophages as PE_PGRS30 [17], −41 [52], and −47 [53]. Most importantly, our results provide an experimental evidence of the ability of PE_PGRS3 on the mycobacterial surface to bind PtdIns and cardiolipin molecules, for their direct integration or as a vital source of phosphate for *Mtb* in the lipid-rich environment of foamy macrophages and caseum. This finding provides a functional rationale for the overexpression of PE_PGRS3 under short supply of phosphate, a condition which limits the ability of *Mtb* to synthesize endogenous vital molecules and likely favors a PE_PGRS3-mediated mechanism to steal these molecules from host cells. The ability of PE_PGRS3 to grab essential molecules during key steps of TB pathogenesis may be considered an ace in the hole of *Mtb*, able to persist for years in caseous granulomas and foamy macrophages.

## Materials and methods

### Bacterial strains, media and growth conditions, and electroporation

*Mycobacterium smegmatis* (*Ms*) mc^2^ 155 and *Mycobacterium tuberculosis* (*Mtb*) H37Rv were grown at 37°C in Middlebrook 7H9 broth medium (difco Becton-Dickinson), supplemented with 0.2% glycerol (Sigma-Aldrich), 10% ADC (Becton-Dickinson), and 0.05% Tween 80 (Sigma-Aldrich) using standard procedures [54,55]. *Ms* and *Mtb* recombinant strains were obtained by electroporating competent cells with above mentioned homemade pMV modified vector containing *hbha* promoter upstream and the sequence coding HA epitope downstream the full-length *Rv0278* gene or its chimeras, respectively. Furthermore, the modified pMV vector carried also the GFP sequence under the control of mycobacterial antigen 85 promoter (**Supplementary Table 1**) [19].

Briefly, *Ms* mc^2^ 155 and *Mtb* H37Rv grown to mid-exponential (log) phase and pre-treated with 1% glycine were widely washed with cold and sterile 10% glycerol. Two hundred microliters of concentrated cells were mixed with 1 μg of DNA, incubated for 5 min (m) at room temperature (RT), and then transferred to 0.2 cm cuvettes (BioRad). Samples were electroporated using an electroporation system Bio-RAD GenePulser X Cell™ with the following parameters: voltage 2500, capacitance 25µF, and resistance 1000 Ω [54]. After the pulse, cells were recovered in 1 ml of 7H9 liquid medium and incubated for 1 day at 37°C.

Colonies were selected on 7H11 agar media supplemented with 10% OADC (Microbiol) containing 40 µg/ml kanamycin (Sigma Aldrich). Single distinct antibiotic-resistant colonies were isolated and sub-cultured in 7H9 medium supplemented with 10% ADC (Microbiol), 0.05% Tween 80, and kanamycin as previously indicated and were incubated at 37°C until mid-log phase. Finally, mycobacteria were stocked at −80°C after adding 20% glycerol until use. Serial dilutions were then carried out to establish bacterial concentration.

### Cloning, expression, and purification of the PE_PGRS3 C-terminal domain

Sequence codifying the last 80 amino acids of the PE_PGRS3 (xR-3Ct) was amplified by using the following primers: Forward: 5ʹ- GGTTGCCGGCGGGTTTGGCGC −3ʹ and Reverse: 5ʹ- CTACGGCATCATCTGCGGTGA −3ʹ. The purified amplicon was cloned in the expression vector pET-SUMO (Invitrogen) downstream to the SUMO – 6x Histidine tag (6xHis) domain and under the control of the *lacO* promoter [20]. Ligation was directly used to transform *E. coli* MACH 1 (Life technologies) and then plated on Luria Bertani agar medium (Sigma Aldrich) supplemented with 50 μg/ml kanamycin (Sigma Aldrich) to select transformant single colonies. Antibiotic-resistant colonies were sub-cultured in Luria Bertani broth medium (Sigma Aldrich) and extracted final vector was controlled by enzymatic restriction analysis and by sequencing.

To express xR-3Ct, *E. coli* BL21-DE3 *pLyss* chemically competent cells (Life technologies) were transformed with pET-SUMO^xR−3Ct^ and then plated on Luria Bertani agar medium (Sigma Aldrich) containing 50 μg/ml Kanamycin (Sigma Aldrich). Selected single colonies were inoculated in Luria Bertani broth medium (Sigma Aldrich) containing 50 μg/ml Kanamycin (Sigma Aldrich), 50 μg/ml chloramphenicol (Sigma Aldrich) supplemented with 1% glucose (Sigma Aldrich), and incubated overnight at 37 C°. 250 ml culture was prepared and incubated until OD_600_ reached 0.6 when 1 mM Isopropyl β-d-1-thiogalactopyranoside (IPTG) (3 V chimica) was added. Three hours post induction, the cell pellet was harvested, washed with sterile cold Phosphate buffer (PBS), and resuspended in lysis buffer (PBS, containing 350 mM NaCl and the protease inhibitor cocktail (Sigma Aldrich), pH: 7.4). Cells were then lysed by sonication. The soluble fraction was collected and processed to purify the xR-3Ct by using Ni-NTA agarose column and Fast Protein Liquid Chromatography (FPLC- ӒKTA, GE health care life sciences) [20,55]. Protein elution was obtained by using PBS containing 350 mM NaCl and 200 mM Imidazole (Sigma Aldrich). rmHBHA was obtained as described above [55].

Purified xR-3Ct and rmHBHA were dialyzed in sterile LPS free PBS by using Float-A-Lyzer G2 columns at 4°C according to the manufacturer’s instruction. Endotoxin was measured by using *Limulus Amebocyte Lysate* assay (LONZA). An endotoxin-free batch was considered at LPS concentration <0.1 EU/ml.

### Lipids/PE_PGRS3 C-terminal domain interaction

Purified xR-3Ct was used to probe a nitrocellulose strip (Echelon) where different phosphorylated lipids were adsorbed [25]. Following lipids were imbedded on the membrane strip: triglyceride (GT), diacylglycerol (DAG), phosphatidic acid (PA), phosphatidylserine (PS), phosphatidylethanolamine (PE), phosphatidylcholine (PC), phosphatidylglycerol (PG), cardiolipin (CL), phosphatidylinositol (PtdIns), PtdIns(4)P, PtdIns(4,5)P2, PtdIns(3,4,5)P3, cholesterol, sphingomyelin (SM), or sulfatide. Nonspecific binding was blocked by incubation with PBS containing 0.1% Tween 20 (PBS-T) and 3% BSA for 1 hour at room temperature. xR-3Ct was added at a final concentration of 0.5 µg/ml and incubated for 1 hour as indicated by the manufacturer’s instruction. The membrane was washed with PBS-T and a monoclonal anti-His antibody (Sigma-Aldrich) was to detect the binding. Finally, an IgG-Peroxidase (Sigma-Aldrich) was used as the secondary antibody. Immunoblot was developed using Supersignal West Dura Extended Duration Substrate (Thermo scientific) and chemiluminescence detected by ChemiDoc TM XRS+ system (Biorad). The same experiment was performed with the rmHBHA as previously described.

### FACS analysis

Recombinant *Ms*, expressing PE_PGRS3 under control of its own promoter and fused at the C-terminal with the GFP (*Ms*PE_PGRS3^GFP^), *Ms* expressing GFP (*Ms*^GFP^) and *Ms* mc^2^ 155 non-fluorescent wild type strain were grown in standard and low phosphate Sauton medium (~50 μM P_i_) as described above. Fluorescence was measured by using FACSCantoII flow cytometer (BD Bioscience), as described elsewhere [19]. When fluorescence of *Ms*PE_PGRS3^GFP^ grown in low P_i_ environment switched on, the liquid culture was split into different tubes. Inorganic phosphate (P_i_), diacylglycerol (DAG), phosphatidic acid (PA), phosphatidylserine (PS), cardiolipin (CL), phosphatidylinositol (PtdIns), PtdIns(4,5)P2, PtdIns(3,4,5)P3, and cholesterol were added at the final concentration of 100 µM. Fluorescence was measured at different time points following phosphate or lipid restoring.

Careful cytometry analysis provided for a gating strategy composed of different and serial steps. Initial Forward scatter (FSC-A) versus Side scatter (SSC-A) was carried out to identify bacteria, based on size and complexity, and to exclude debris. Progressively, two serial gating were carried out to get out doublets or inappropriate heterogeneities. Forward scatter height (FSC-H) versus FSC-A density plot and a Side scatter height (SSC-H) versus SSC-A plot were opportunely performed before measuring fluorescence. The fluorescence intensity of at least 50,000 ungated events was measured as previously described [19]. The excitation laser line was at 488 nm (Excitation max value was 494 nm and emission max value was 520 nm). The data files were analyzed using *FACSDiva Software* (BD Bioscience).

### Malachite green assay

Phosphatase activity of the xR-3Ct was assessed by using a malachite green based phosphatase assay (Echelon). Free P_i_ in solution forms a colored complex with molybdate/malachite green that is quantified by reading absorbance at 620 nm. The following phosphorylated phosphatidylinositols (PtdIns) were assayed: PtdIns(3)P, PtdIns(4)P, PtdIns(5)P, PtdIns(3,4)P, PtdIns(3,5)P, PtdIns(4,5)P, PtdIns(4,4,5)P.

Briefly, 25 μl containing 5 μg/ml of the xR-3Ct and rmHBHA were mixed with 25 μl of the PtdIns and incubated 1 h at 37°C in agitation. After incubation, 100 μl of room temperature malachite green solution was added to each well and the plate was incubated for 30 minutes without shaking. Absorbance was measured as above indicated.

Phosphatase activity of the xR-3Ct was also assessed on recombinant *Ms* strains expressing full-length PE_PGRS3 (*Ms^GFP^PE_PGRS3^HA^*) or its chimera lacking CT domain (*Ms^GFP^PE_PGRS3ΔCT^HA^*) and *Ms^GFP^*. In the culture supernatant was measured free P_i_ (data not shown).

### Zeta potential measurements

Recombinant *Ms* overexpressing PE_PGRS3 (*Ms^GFP^PE*_*PGRS3^HA^*), its functional mutant lacking C-terminal domain (*Ms^GFP^PE_PGRS3ΔCT^HA^*) and wild type (*Ms^GFP^*) strains were grown in 7H9 completed medium until mid-log phase and then sub-inoculated in Sauton standard medium supplemented with 40 µg/ml kanamycin. Each specimen was diluted in water (1:100) and incubated with PtdIns(4)P, PtdIns(4,5)P2, PtdIns(3,4,5)P3 for 1 h. Finally, solutions were characterized by Zetasizer Nano ZS (Malvern, Herrenberg, Germany) equipped with a 633 nm He–Ne laser. Universal zeta dip cell (ZEN1002, Malvern, Herrenberg, Germany) was used for experiments with a sample volume of 1 ml. For each sample, three measurements were averaged. The Z-potential was calculated from the electrophoretic mobility using the Henry correction to Smoluchowski’s equation as reported previously [19].

### Proteinase K treatment

Recombinant *Mtb* strains were cultured in 7H9 medium supplemented with 10% ADC and 0.05% Tween 80, before sub-inoculating in Sauton medium until mid-log phase. Cells were harvested by centrifugation and washed two times with cold sterile PBS. Each pellet was re-suspended in the Buffer G2 (Qiagen) and divided into two aliquots. Proteinase K (Qiagen) was added to one of the samples according to the manufacturer’s instruction. Both samples were incubated in agitation for 30 minutes at RT. Finally, each aliquot was washed three times with PBS and resuspended in lysis buffer as previously described [19].

### Mycobacterial cell lysis, SDS-PAGE, western blotting, and immunoblotting

Recombinant *Mtb* strains were cultured as previously described and then sub-inoculated in diverse Sauton based medium until mid-log phase when cells were harvested by centrifugation (3500 rpm for 15 minutes, 4 C°). Growth conditions account for Sauton medium combinations supplemented or not with inorganic phosphate (P_i_) or with 0.05% Tween 80. Each culture was divided into two aliquots. To obtain whole cell lysate (WCL), the cell pellet was washed with cold sterile PBS and re-suspended in lysis buffer (20 mM Tris, 150 mM NaCl, 1:100 protease inhibitor cocktail (Sigma Aldrich), pH: 7.5). Finally, the lysate was obtained using a mini bead beater (BioSpec) instrument [34,54]. To separate mycomembrane associated proteins, the cell pellet of the second aliquot was treated with lysis buffer containing 0.5% Genapol X-80 (Fluka) for 30 minutes at room temperature. The supernatant (Genapol fraction) was collected by centrifugation and pellet (Cytosolic fraction) was washed with cold PBS before lysis as previously described. Finally, secreted proteins were obtained by TCA precipitation of the culture medium. Proteins were separated on 4– 15% precast polyacrylamide gel (Smobio) by SDS-PAGE and then transferred to a nitrocellulose membrane (Bio-Rad) by western blotting. Nonspecific protein binding was blocked by treatment with PBS containing 0,05% Tween 20 (Sigma Aldrich) and 5% Skim Milk (Oxoid) for 1 h at room temperature.

Membranes were probed with monoclonal anti-HA (1:1000) (Covance), monoclonal anti-GFP (1:4000) (Sigma Aldrich), polyclonal anti-GroEL (1:5000) (GeneTex) antibodies, and with pooled sera (1:2000) obtained from mice immunized with the purified MPT64 antigen. IgG-Peroxidase (Sigma Aldrich) was used as a secondary antibody. Immunoblot was developed using Supersignal West Dura Extended Duration Substrate (Thermo scientific), and finally, chemiluminescence was detected by ChemiDoc TM XRS+ system (Biorad).

### Capture of the PE_PGRS33 ^HA^ in the mycobacterial cell wall

*Mtb* and *Mycobacterium bovis* (*Mbov*) BCG were expressing the PE_PGRS33. Recombinant strains were obtained by cloning the *Rv1818c* gene, with its putative promoter, upstream the HA epitope sequence in a pMV206-based vector [31,32]. Recombinant *MtbPE_PGRS33 ^HA^* and *MbovPE_PGRS33 ^HA^* and the parental strains *Mtb* and *Mbov* BCG were grown as previously described and then sub-inoculated in Sauton medium, supplemented or not with 0.05% Tween 80, until mid-log phase (OD_600 nm_: 0.8 ± 0.2). Cultures were harvested and processed to obtain the Genapol fraction as indicated above. Genapol fractions of all strains were incubated with anti-HA magnetic beads (Miltenyi Biotec) following the manufacturer’s instruction. Beads binding PE_PGRS33 ^HA^ were separated from the solution by using a magnetic plate, washed three times before eluting the captured protein. Eluates were TCA precipitated before protein separation by SDS page electrophoresis and immunoblot as previously described. The membrane was probed with the anti-HA (1:1000) monoclonal antibody and anti-GroEL polyclonal antibody (1:5000).

### Cell culture and mycobacteria infection

Murine macrophages (J774 – A1) and human type 2 pneumocytes (A549) were grown in Dulbecco’s modified eagle medium (DMEM) (Euroclone) enriched with 10% fetal bovine serum (FBS), 2 mM glutamine (Euroclone), 100 µg/ml streptomycin, and penicillin (Euroclone) and were kept in a humidified atmosphere containing 5% CO_2_ at 37°C. Before infection, cells were collected and suspended in the same medium without antibiotic and supplemented with 2% FBS. Cells were plated at the concentration of 1.2 × 10^6^ cell/ml and were infected 24 h later [19,54].

Multiplicity of infection (MOI) 10:1 for 4 h was used for *Ms* infection for both J774 and A549; conversely an MOI of 1:1 (J774) and MOI of 10:1 (A549) for 1 h was used for *Mtb* infection and cells were maintained at standard atmosphere conditions (5% CO_2_ and 37°C) [32]. Intracellular colony forming units (CFUs) were obtained at 4 h post infection for *Ms* and 1 h post infection for *Mtb* infected cells.

For the infection with *Ms*, xR-3Ct and rmHBHA were added to the infection solution at the final concentration of 1 μg/ml [20].

Peripheral blood mononuclear cells (PBMCs) were isolated from human blood collected by healthy volunteers by using Ficoll lympholyte (Cederlane) following the manufacturer’s procedure. PBMCs were washed with sterile PBS and then resuspended in Roswell Park Memorial Institute (RPMI) 1640 medium (Euroclone) enriched with 10% FCS and 2 mM glutamine (Euroclone). Finally, PBMCs were seeded at a final concentration of 1.2 × 10^6^ cell/ml and infected with *Mtb* recombinant strains at MOI = 1:10 [56]. CFUs evaluated at 1 and 7 days post infection.

## Statistical analysis

All data were generated from independent experiments with at least three technical replicates. *Microsoft Excel* (2010) and *Graphpad Prism* software version 6 (GraphPad software) were used to collect and to analyze the data. Data were expressed on a representative graph as mean ± SD and analyzed by one-way or two-way ANOVA comparison tests followed by the appropriate correction, as specified in the caption under each figure.

### Modeling of xR-3Ct structure

Secondary structure prediction of xR-3Ct was performed using JPRED4 server [57]. Homology modeling was performed using I-Tasser [58]. A meta-threading approach, LOMETS, was used to retrieve template proteins of similar folds from the PDB library. The program SPICKER was used to cluster the decoys based on the pair-wise structure similarity. Best confidence model was selected based on the C-score. The electrostatic potential surface was computed with Chimera [59].

## Supplementary Material

Supplemental Material

## Data Availability

Raw data were generated at Università Cattolica del Sacro Cuore and Fondazione Policlinico Gemelli (Dipartimento di Scienze biotecnologiche di base, cliniche intensivologiche e perioperatorie – Sezione di Microbiologia; Dipartimento di Diagnostica per Immagini, Radioterapia Oncologica ed Ematologia; Dipartimento di Neuroscienze, Università Cattolica del Sacro Cuore) and Institute of Biostructures and Bioimaging. Derived data supporting the findings of this study are available from the corresponding author GD on request.
