# Supplementary material for: PE_PGRS3 ensures provision of the vital phospholipids cardiolipin and phosphatidylinositols by promoting the interaction between M. tuberculosis and host cells
Source: Virulence. 2021 Mar 23;12(1):868–84. doi: 10.1080/21505594.2021.1897247 (PMC8007152; doi:10.1080/21505594.2021.1897247)
Supplement: Supplemental Material [file KVIR_A_1897247_SM7177.zip › cap.Document.rtf]

Supplementary Figure 1: Expression and purification of the arginine rich C-terminal domain of the PE_PGRS3. Last 240 base pairs representing the 80 aa form the arginine rich C-terminal domain of the Rv0278c gene (xR-3Ct) was amplified and cloned in frame with the histidine tagged SUMO epitope in a pET-based expression vector (A). Recombinant peptide (SUMO-CT: ~ 10kDa + ~ 10kDa, respectively) was expressed in E. coli and purified by using FPLC (B): fraction 1 (Elution buffer – 100mM Imidazole) and fraction 2 (Elution buffer – 200mM Imidazole). Elution fractions were firstly probed with anti-HIS monoclonal antibody (C), and then mixed before carrying out the dialysis in PBS and LPS removal: lane 1 and 2 respectively (D).

Supplementary Figure 2: xR-3Ct and rmHBHA lipid binding. Nitrocellulose membranes with phosphorylated lipids probed with positive charged arginine rich C-terminal domain of the PE_PGRS3 (xR-3Ct) (A) and Mtb heparin-binding hemagglutinin (rmHBHA), which contains positive charged lysins (B). TG: Triglyceride, DAG: Diacylglycerol, PA: Phosphatidic acid, PS: Phosphatidylserine, PE: Phosphatidylethanolamine, PC: Phosphatidylcholine, PG: Phosphatidylglycerol, CL: Cardiolipin, PI: Phosphatidylinositol, PtdIns(4)P: Phosphatidylinositol 4 phosphate, PtdIns 4,5(P): Phosphatidylinositol 4,5 phosphate, PtdIns (3,4,5)P: Phosphatidylinositol 3,4,5 phosphate, C: Cholesterol, SM: Sphingomyelin, S: Sulfatide. Data are representative of a single experiment repeated three times.

Supplementary Figure 3: Chemical structures of all tested phospholipids and their respective charge.

Table S1: List of the mycobacterial strains used in this work.
Strain		Gene reporter		PE_PGRS	
	 		 	promoter	gene	epitope	
MsGFPPE_PGRS3HA	 	GFP	 	hbha	Rv0278c	HA	
MsGFPPE_PGRS∆CTHA	 	GFP	 	hbha	Rv0278c∆CT	HA	
MsGFP	 	GFP	 	-	-	-	
MsPE_PGRS3GFP		-		Rv0278c	Rv0278c	GFP	
Ms mc2 155		-		-	-	-	
MtbGFPPE_PGRS3HA	 	GFP	 	hbha	Rv0278c	HA	
MtbGFPPE_PGRS∆CTHA	 	GFP	 	hbha	Rv0278c∆CT	HA	
MtbGFPPE_PGRS∆GRPLIHA	 	GFP	 	hbha	Rv0278c∆GRPLI	HA	
MtbGFP	 	GFP	 	-	-	-	
MtbPE_PGRS33HA		-		Rv1818c	Rv1818c	HA	
M.bovis BCG-PE_PGRS33HA		-		Rv1818c	Rv1818c	HA	
Mtb H37Rv		-		-	-	-	
MbovBCG		-		-	-	-	
