# Supplementary figures and images for: PE_PGRS3 ensures provision of the vital phospholipids cardiolipin and phosphatidylinositols by promoting the interaction between M. tuberculosis and host cells
Source: Virulence. 2021 Mar 23;12(1):868–84. doi: 10.1080/21505594.2021.1897247 (PMC8007152; doi:10.1080/21505594.2021.1897247)

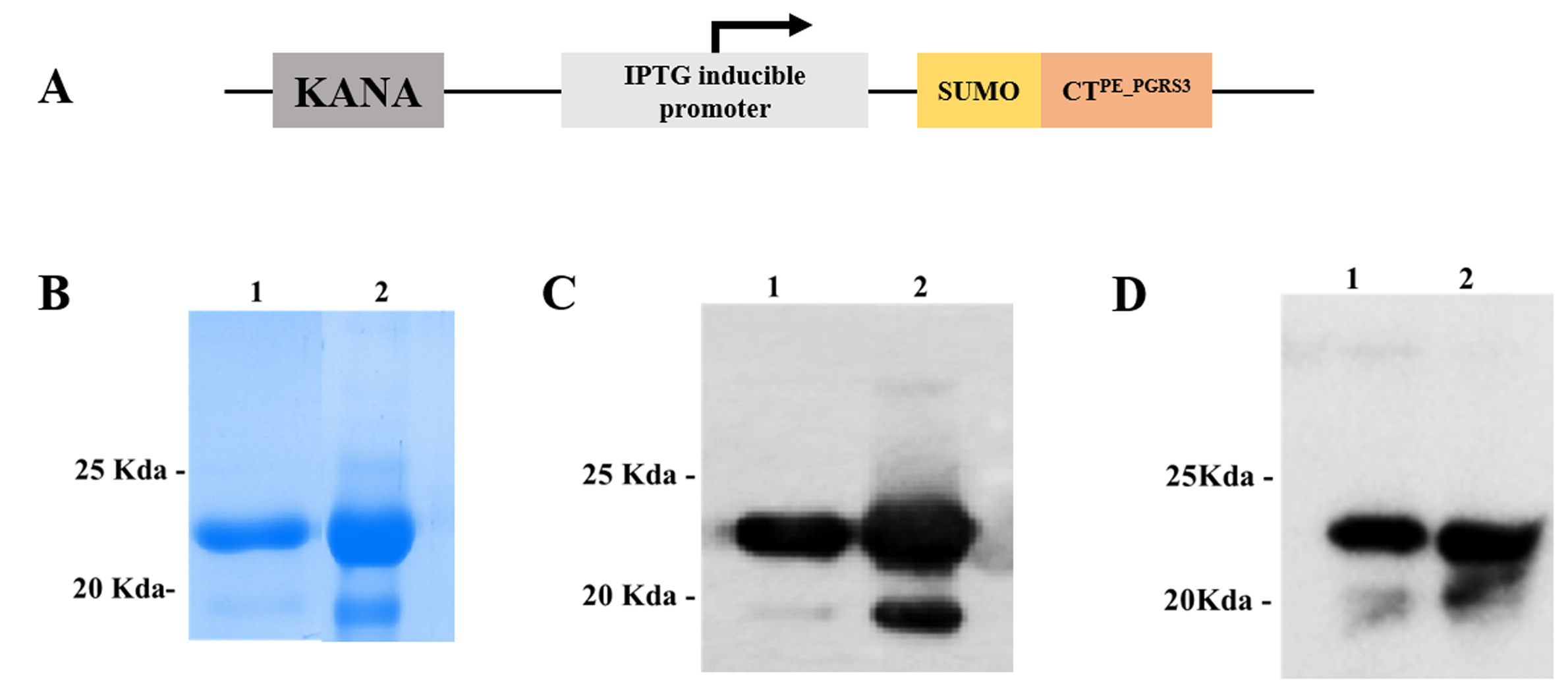

Supplement: Supplemental Material [file KVIR_A_1897247_SM7177.zip › Supplementary Figure 1.tif]

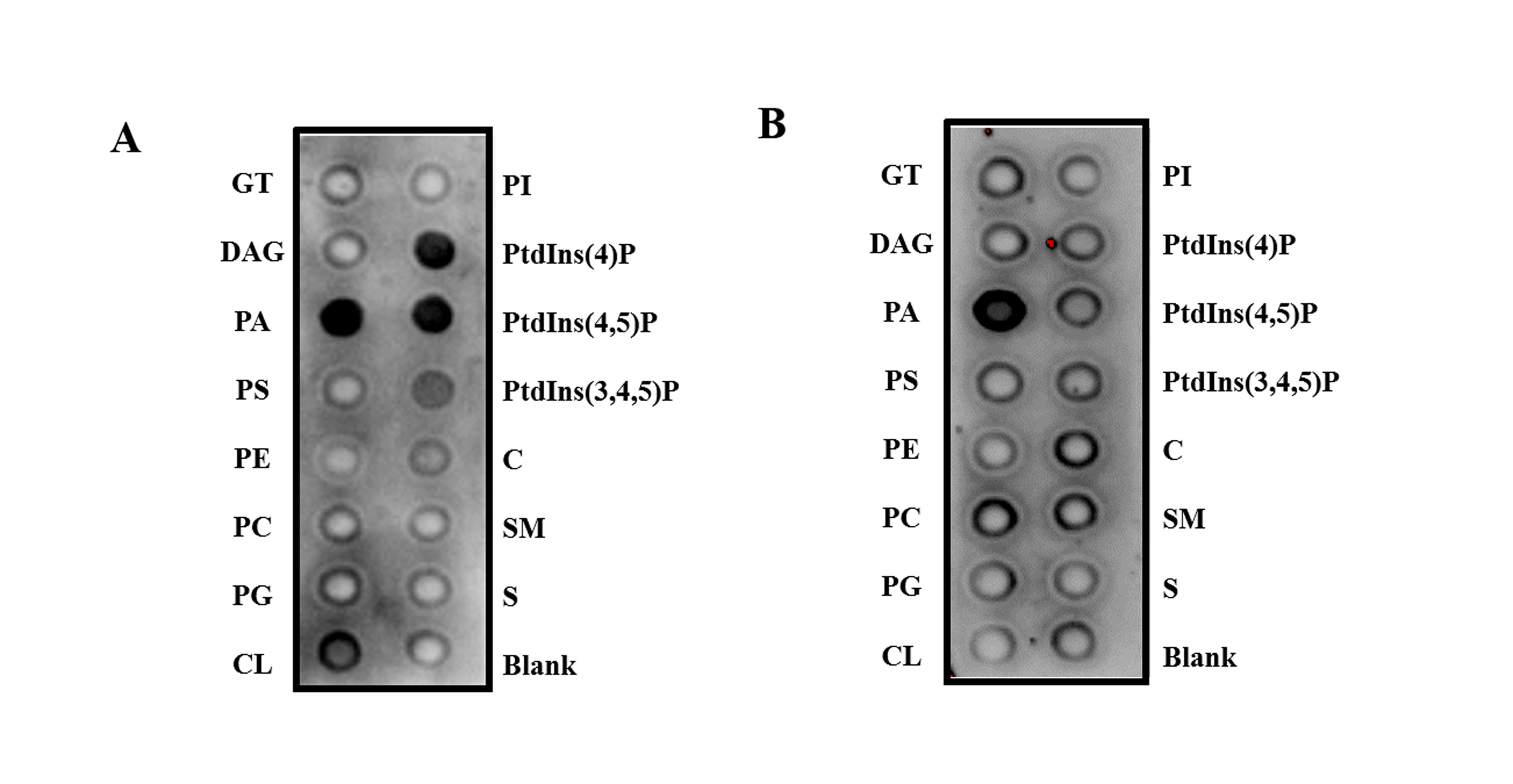

Supplement: Supplemental Material [file KVIR_A_1897247_SM7177.zip › Supplementary Figure 2.tif]

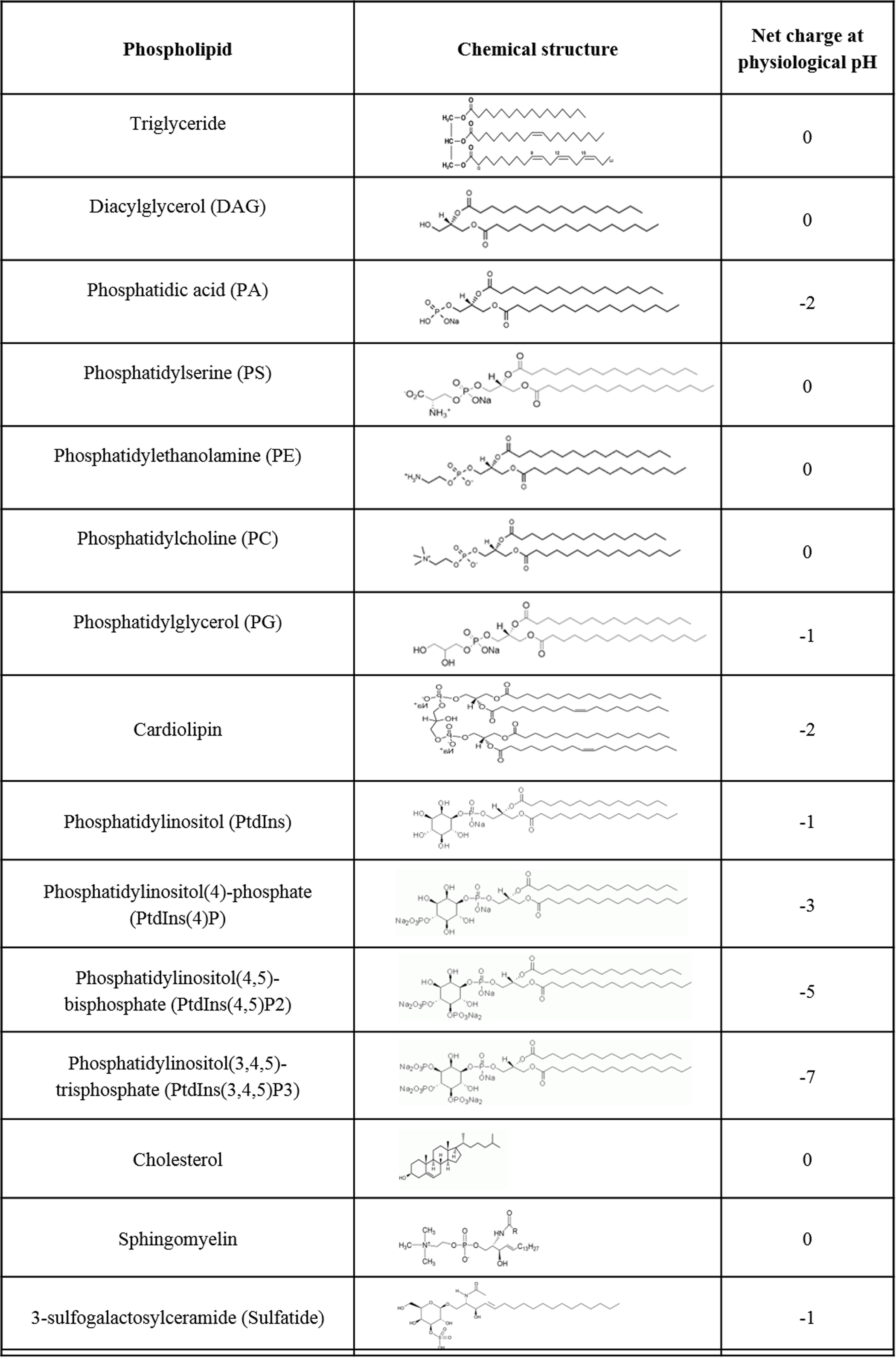

Supplement: Supplemental Material [file KVIR_A_1897247_SM7177.zip › Supplementary Figure 3.tif]
